# CA19-9 in combination with P-CRP as a predictive marker of immune-related adverse events in patients with recurrent or unresectable advanced gastric cancer treated with nivolumab

**DOI:** 10.1186/s12885-022-09482-8

**Published:** 2022-04-15

**Authors:** Tomoyuki Matsunaga, Hiroaki Saito, Hirohiko Kuroda, Tomohiro Osaki, Sadamu Takahashi, Akemi Iwamoto, Yoji Fukumoto, Kenjiro Taniguchi, Kenji Fukuda, Wataru Miyauchi, Yuji Shishido, Kozo Miyatani, Yoshiyuki Fujiwara

**Affiliations:** 1 Division of Gastrointestinal and Pediatric Surgery, Department of Surgery, School of Medicine, Faculty of Medicine, Tottori University, Yonago, 683-8504 Japan; 2 Department of Surgery, Japanese Red Cross Tottori Hospital, Tottori, 680-8517 Japan; 3 Department of Surgery, Japanese Red Cross Masuda Hospital, Masuda, 698-8501 Japan; 4 Department of Surgery, Tottori Prefectural Central Hospital, Tottori, 680-0901 Japan; 5 National Hospital Organization, Hamada Medical Center, Hamada, 697-8511 Japan; 6 Divisions of Digestive Surgery, Tottori Prefectural Kousei Hospital, Kurayoshi, 682-0804 Japan; 7 Division of Gastroenterology, Matsue City Hospital, Matsue, 690-8509 Japan; 8 Department of Surgery, Yonago Medical Center of National Hospital Organization, Yonago, 683-0006 Japan; 9 Department of Surgery, Sanin Rosai Hospital, Yonago, 683-8605 Japan

**Keywords:** Gastric cancer, Immune-related adverse events, Nivolumab, Prognosis

## Abstract

**Background:**

Immune-check point inhibitors (ICPIs) for treatment of cancer patients sometimes induce potentially life-threatening immune-related adverse events (irAEs), which predict ICPIs treatment efficacy. Prediction of irAEs would be useful for management of irAEs and prediction of ICPIs efficacy. This study aimed to determine predictors of irAEs in patients with recurrent or unresectable advanced gastric cancer (RUGC) treated with nivolumab.

**Methods:**

Seventy-eight RUGC patients treated with nivolumab at nine institutions between January 2017 and April 2020 were included in this study. The usefulness of specific blood test results as predictors of irAEs was evaluated.

**Results:**

We observed irAEs in 15 (19.2%) patients. The disease control rate was significantly higher in the patients with irAEs than in those without (86.7% vs. 42.9%; *P* < 0.001). The median progression-free survival was significantly longer for patients with irAEs than for patients without (4.9 vs. 2.6 months; *P* = 0.018). The median survival time was longer for patients with irAEs than for those without (9.4 vs. 5.8 months; *P* = 0.041). The receiver operating characteristic (ROC) curves for irAEs indicated that the area under the curve (AUC) of carbohydrate antigen 19–9 (CA19-9) was highest (0.692; *P* = 0.022), followed by that for the platelet count × serum C-reactive protein (P-CRP) value (0.680; *P* = 0.032). The AUC for the CA19-9 + P-CRP combination was 0.782, which was more useful than that for either component and significantly associated with overall survival of nivolumab-treated RUGC patients.

**Conclusions:**

The CA19-9 + P-CRP combination was predictive of irAEs and prognosis in RUGC patients.

## Background

Gastric cancer (GC) is the fourth most common cancer and second leading cause of cancer-related deaths worldwide [1, 2]. Recent advances in chemotherapy have prolonged survival in patients with recurrent or unresectable advanced GC (RUGC) [3–5]. Furthermore, the development of immune-check point inhibitors (ICPIs) has significantly improved the prognosis of patients with various types of cancer, including GC [6]. Nivolumab is an ICPI that is a fully humanized immunoglobulin G4 anti-programmed cell death (PD)-1 antibody. Since it disrupts PD-1-mediated signaling, which negatively regulates T-cell function, nivolumab enhances antitumor immunity and shows antitumor activity [7, 8]. In fact, the ATTRACTION 2 study, which was a double-blind, placebo-controlled, randomized, phase 3 trial, has shown improvement of overall survival (OS) in RUGC patients who are refractory to or intolerant of two or more previous regimens of chemotherapy [6]. However, nivolumab treatment efficacy was not observed in approximately 60% of the patients in the trial. Hyperprogressive disease (HPD) was recently observed in various types of cancer, including nivolumab-treated RUGC patients [9]. Furthermore, it has been shown recently that trifluridine/tipiracil was effective for treatment of patients with heavily pretreated metastatic GC [10]. Therefore, it is quite important to select patients in whom nivolumab treatment is likely to be effective. To this end, there is a strong need to develop useful predictors of nivolumab treatment efficacy.

ICPIs sometimes induce immune-related adverse events (irAEs). Most irAEs induced by ICPIs are mild and reversible if they are diagnosed early and properly managed. However, some irAEs, such as serious colitis, pneumonia, and myocarditis, are even more life-threatening [11, 12]. Importantly, those irAEs are not observed in conventional anti-cancer drug treatments, and they sometimes require treatment by a specialist who may use steroids and other immunosuppressive agents. Therefore, it is quite important to have predictive indicators of irAEs for early detection and treatment for irAEs.

Accumulating evidence has indicated that the prognosis of patients with irAEs was significantly better than that of patients without irAEs [13, 14], indicating that presence of irAEs could be useful prognostic indicators. However, it is impossible to predict irAEs before initiation of nivolumab treatment. Considering the close correlation between irAEs and prognosis in nivolumab-treated cancer patients, we speculated that development of predictors of irAEs in nivolumab treatment might be useful for both irAEs management and prediction of RUGC patient prognosis. Therefore, the study aim was to determine predictors of irAEs in patients with RUGC treated with nivolumab.

## Methods

### Patients

**Fig. 1:**
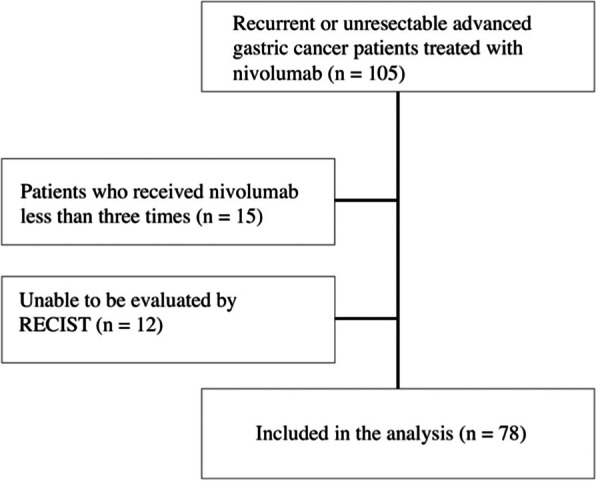
CONSORT diagram CONSORT, Consolidated Standards of Reporting Trials

A total of 105 patients with RUGC who underwent nivolumab treatment at nine institutions between January 2017 and April 2020 were enrolled in this study. The clinicopathological findings were determined according to the Japanese GC treatment guidelines [15]. Clinical data, including age, sex, Eastern Cooperative Oncology Group (ECOG) performance status (PS), histology, HER-2 status, and metastatic site at the time of starting nivolumab treatment were collected from the databases of the nine hospitals. Among the 105 patients enrolled in this study, either the patients in whom the treatment efficacy could not be evaluated by the Response Evaluation Criteria in Solid Tumors (RECIST) or those who received nivolumab less than three times were excluded. As a result, a total of 78 patients were included in the analysis (Fig. 1). Details of the patients included in this study are presented in Table 1. The study protocol was approved by the institutional review board of each participating hospital.

**Table 1:** Clinicopathological features of RUGC patients included in this study

Age	62 (38–88)
Sex (male/female)	59/19
ECOG PS (0 / 1 / 2 / 3)	29 / 37 / 10 / 2
Disease status (unresectable / recurrence)	41 / 37
Histology (differentiated / undifferentiated / unknown)	45 / 31 /2
HER2 status (positive / negative / unknown)	17 / 46 / 15
Number of previous treatments (2 / 3 / 4)	65 / 8 / 5
Site of metastasis or recurrence	
Peritoneal metastasis	41
Lymph-node metastasis	23
Liver metastasis	18
Bone metastasis	5
Others	11
Number of nivolumab treatments	12 (3–40)
irAEs (present / absent)	15 / 63

Data are presented as the median (min–max) or number (percentage) of patients

*RUGC* Recurrent or unresectable advanced gastric cancer, *ECOG* Eastern Cooperative Oncology Group, *PS* Performance status, *irAEs* immune-related adverse events

### Treatment and assessment

Nivolumab was administered intravenously at a dose of either 3 mg/kg or 240 mg/body every 2 weeks. At that time, patients underwent physical examination and blood tests for evaluation of adverse effects. Abdominal ultrasonography and/or computed tomography were performed every 6 to 8 weeks. RECIST version 1.1 was used to evaluate the treatment efficacy. Adverse effects were evaluated according to the National Cancer Institute Common Terminology Criteria for Adverse Events ver. 4.03. No patients included in this study underwent other treatment for GC, such as chemotherapy, radiation therapy, and other immunotherapy, during nivolumab treatment.

### Predictive indicators for irAEs

Peripheral neutrophil count (NC), lymphocyte count (LC), platelet count (PC), serum albumin (ALB; g/dl) level, lactate dehydrogenase (LDH; IU/l), carcinoembryonic antigen level (ng/ml), and carbohydrate antigen 19–9 (CA19-9) level (U/ml) were measured at the initiation of nivolumab treatment. The neutrophil-to-lymphocyte ratio and platelet-to-lymphocyte ratio (PLR) were calculated by dividing either peripheral NC or PC by the peripheral LC, respectively. The prognostic nutritional index (PNI) was calculated by using the following formula: PNI = 10 × ALB concentration + 0.005 × total LC [16]. The C-reactive protein (CRP)/ALB ratio was calculated by dividing the serum CRP level by the serum ALB level. The PC × serum CRP level multiplier value (P-CRP) was calculated according to the following formula: P-CRP = peripheral PC × serum CRP level / 10^4^ [17].

### Statistical analysis

Continuous variables are expressed as the mean and compared by using the Mann–Whitney U test. The χ^2^ test or Fisher’s exact test was used to compare categorical variables. Receiver operating characteristic (ROC) analysis was used to determine the Youden index and area under the curve (AUC) for irAEs. Progression-free survival (PFS) was defined as the time from initiation of nivolumab treatment to the date of disease progression or the date of death from any cause. OS was measured until death or censoring at the latest follow-up for surviving patients. Survival curves were calculated by using the Kaplan–Meier method, and differences between survival curves were examined by using the log-rank test. Values of *P* < 0.05 were considered to be indicative of statistical significance. All statistical analyses were performed by using IBM SPSS Statistics for Windows, Version 25 (IBM Corp., Armonk, NY).

## Results

### Response to nivolumab treatment and prognosis

**Fig. 2:**
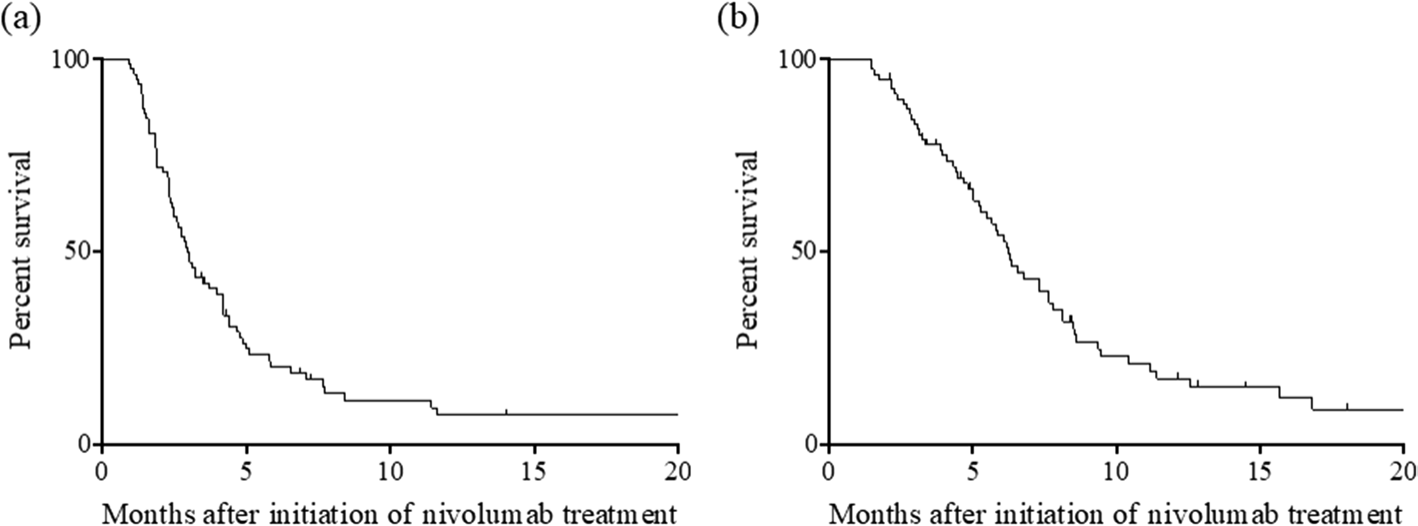
Progression-free survival curves (**a**) and overall survival curves (**b**) in RUGC patients treated with nivolumab RUGC, recurrent or unresectable advanced gastric cancer

In this study, 2 (2.6%) patients achieved a complete response, 8 (10.2%) achieved a partial response, and 18 (37.2%) achieved stable disease, with the remaining 54 patients experiencing progressive disease (PD) (50.0%). The objective response rate (ORR) and disease control rate (DCR) were 12.8% (10 of 78 patients) and 50.0% (39 of 78 patients), respectively (Table 2). The median follow-up period was 5.4 months (range, 2.7–18.1 months), and 56 (71.8%) of the 78 patients had died by the time of analysis. The median PFS was 3.0 months [95% confidence interval (CI), 2.432–3.501; Fig. 2a], and the median OS was 6.3 months (95% CI, 5.303–7.231; Fig. 2b).

**Table 2:** Responses to nivolumab treatment

		irAE ( +) (*n* = 15)	irAE ( −) (*n* = 63)
CR	2 (2.6)	1 (6.7)	1 (1.6)
PR	8 (10.2)	3 (20.0)	5 (7.9)
SD	29 (37.2)	9 (60.0)	21 (33.3)
PD	39 (50.0)	2 (13.3)	36 (57.2)
ORR	10 (12.8)	4 (26.7)	6 (9.5)
DCR	39 (50.0)	13 (86.7)	27 (42.8)

*CR* Complete response, *PR* Partial response, *SD* Stable disease, *PD* Progressive disease, *ORR *Objective response rate, (CR + PR) * 100 / total cases *DCR*, Disease control rate, (CR + PR + SD) * 100 / total cases

### Comparison of clinicopathological characteristics between patients with irAEs and those without irAEs

Fifteen (19.2%) patients experienced irAEs in this study. Table 3 shows the details of the irAEs. Although most irAEs were mild (grades 1 and 2 in 80% of the patients), some severe irAEs were also observed: one grade 4 (liver failure) and two grade 3 (colitis). The comparisons of clinicopathological characteristics between the patients with and without irAEs are summarized in Table 4. No significant differences were observed in age, sex, ECOG PS, disease status, histology, HER2 status, and number of previous treatments.

**Table 3:** Details of irAEs observed in this study

	Grade 1	Grade 2	Grade 3	Grade 4	Grade 5	Any grade
Hypothyroidism	1	4	0	0	0	5
Liver	0	0	0	1	0	1
Diarrhea / colitis	2	1	2	0	0	5
Adrenal insufficiency	0	1	0	0	0	1
Rash	0	2	0	0	0	2
Peripheral motor neuropathy	0	1	0	0	0	1
Total	3	9	2	1	0	15

*irAEs*, Immune-related adverse events

**Table 4:** Clinicopathological characteristics of the patients with and without irAEs

	irAEs + (*n* = 15)	irAEs − (*n* = 63)	*P* value
Age	65 (41–81)	67 (38–88)	0.560
Sex			0.116
Male	9 (60.0)	50 (79.4)	
Female	6 (40.0)	13 (20.6)	
ECOG PS			0.701
0/1	13 (86.7)	52 (82.5)	
2/3	2 (13.3)	11 (17.5)	
Disease status			0.947
Unresectable	8 (53.3)	33 (52.4)	
Recurrence	7 (46.7)	30 (47.6)	
Histology			0.142
Differentiated	12 (80.0)	33 (52.4)	
Undifferentiated	3 (20.0)	28 (44.4)	
Unknown	0	2 (3.2)	
HER2 status			0.108
Positive	6 (40.0)	11 (17.5)	
Negative	8 (53.3)	38 (60.3)	
Unknown	1 (6.7)	14 (22.2)	
Number of previous treatments			0.700
2	13 (86.7)	52 (82.5)	
3 / 4	2 (13.3)	11 (17.5)	
Response to nivolumab treatment			< 0.001
CR/PR/SD	13 (86.7)	27 (42.9)	
PD	2 (13.3)	36 (57.1)	

Data are presented as the median (min–max) or number (percentage) of patients

*ECOG,* Eastern Cooperative Oncology Group *PS*, Performance status *irAEs*, Immune-related adverse events; *CR*, complete response *PR*, partial response *SD*, Stable disease *PD*, Progressive disease

### Prognosis according to irAEs

**Fig. 3:**
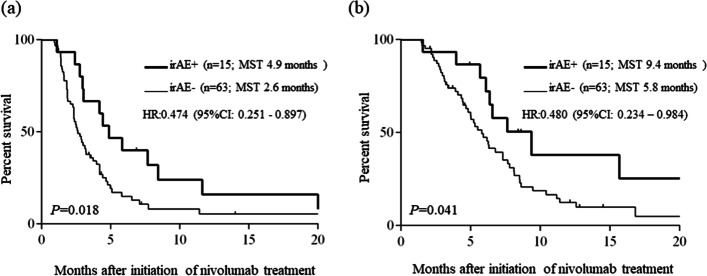
Progression-free survival curves (**a**) and overall survival curves (**b**) according to irAEs in RUGC patients treated with nivolumab irAEs, immune-related adverse events; MST, median survival time; RUGC, recurrent or unresectable advanced gastric cancer

The DCR was significantly higher in the patients with irAEs than in those without irAEs (86.7% vs. 42.9%; *P* < 0.001; Table 4). The median PFS was significantly longer in the patients with irAEs (4.9 months; 95% CI, 2.8–6.9 months) than in those without irAEs (2.6 months; 95% CI, 2.1–3.2 months; *P* = 0.018, Fig. 3a). Furthermore, the median OS was significantly longer in the patients with irAEs (9.4 months; 95% CI, 5.3–13.5) than in those without irAEs (5.8 months; 95% CI, 4.4–7.2; *P* = 0.041, Fig. 3b).

### Development of useful predictors of irAEs by CA19-9 and PCRP

**Fig. 4:**
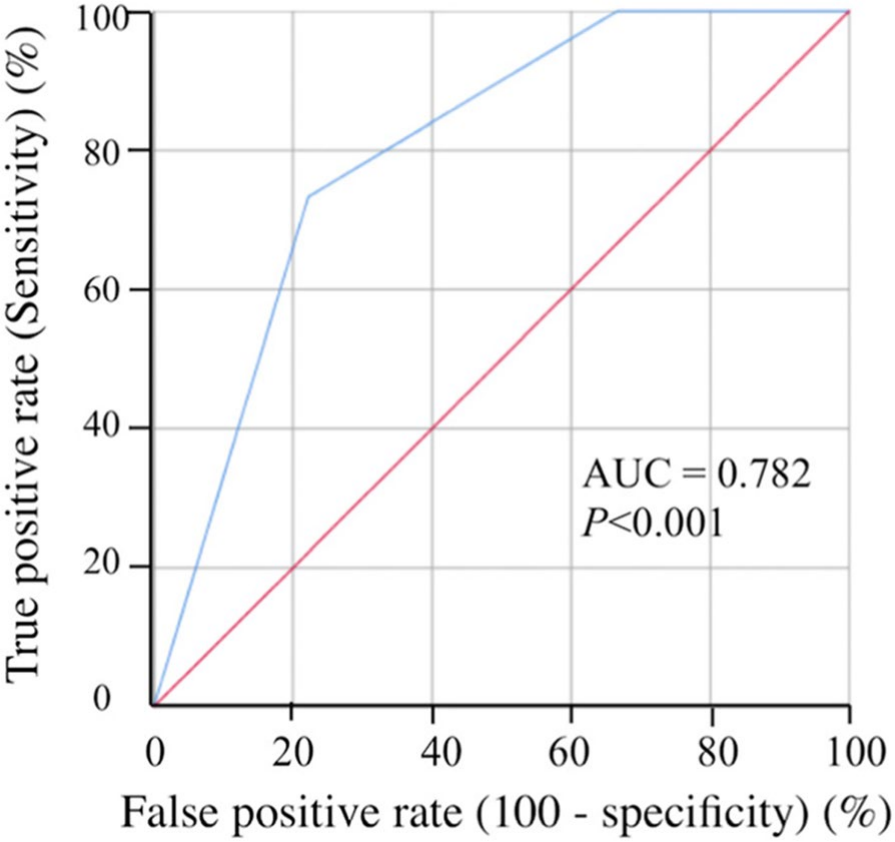
ROC curves of the combination of CA19-9 and P-CRP for the irAEs CA19-9, carbohydrate antigen 19–9; irAEs, immune-related adverse events; P-CRP, platelet count × serum C-reactive protein level multiplier value; ROC, receiver operating characteristic

**Fig. 5:**
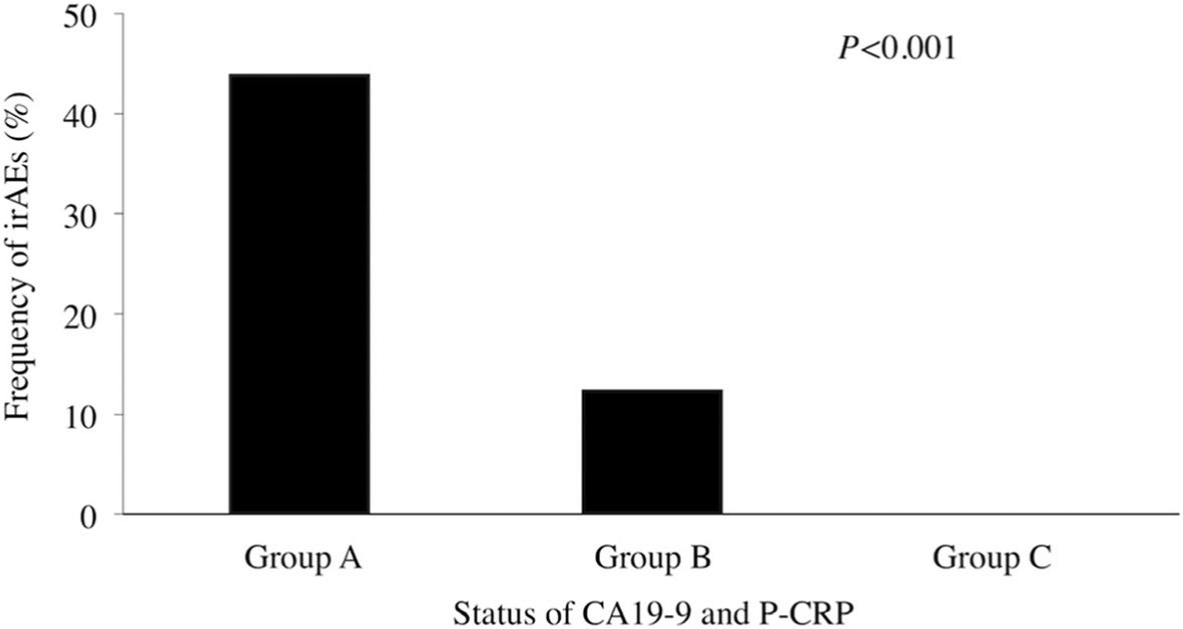
The frequency of irAEs according to the combination of CA19-9 and P-CRP Group A, both CA19-9^Low^ and P-CRP^Low^; Group B, either CA19-9^High^ or P-CRP^High^; Group C, both CA19-9^High^ and P-CRP^High^; CA19-9, carbohydrate antigen 19–9; irAEs, immune-related adverse events; P-CRP, platelet count × serum C-reactive protein level multiplier value; ROC, receiver operating characteristic

We determined the usefulness of 11 peripheral blood markers predictors of irAEs by performing ROC analysis (Table 5). The AUC of CA 19–9 (AUC = 0.692, *P* = 0.022) was the highest, followed by that of P-CRP (AUC = 0.680, *P* = 0.032). ROC analysis showed that the optimal cutoff values of CA19-9 and P-CRP for irAEs were 27.0 and 17.8, respectively. The patients were divided on the basis of these cutoff values as follows: CA19-9^High^ (CA19-9 ≥ 27.0; *n* = 40), CA19-9^Low^ (CA19-9 < 27.0; *n* = 38), P-CRP^High^ (P-CRP ≥ 17.8; *n* = 31), and P-CRP ^Low^ (P-CRP < 17.8; *n* = 47). The irAEs were observed in three (7.5%) patients of CA19-9^High^ and in 12 (31.6%) patients of CA19-9^Low^ (*P* = 0.007). Furthermore, irAEs were observed in one (3.2%) patient with P-CRP^High^ and in 14 (29.8%) patients with P-CRP^Low^ (*P* = 0.004). Since there was a statistically significant but weak correlation between CA19-9 and P-CRP (r = 0.26; *P* = 0.027), we speculated that the combination of CA19-9 and P-CRP was more useful for predicting irAEs than either alone. The patients with both CA19-9^Low^ and P-CRP^Low^ (group A), those with either CA19-9^High^ or P-CRP^High^ (group B), and those with both CA19-9^High^ and P-CRP^High^ (group C) were assigned 0, 1, and 2, respectively [18]. ROC analysis indicated that the AUC of the combination of CA19-9 and P-CRP for irAEs was 0.782, which was much higher than that of either CA19-9 or P-CRP (*P* < 0.001, Fig. 4). The rates of irAEs were 44.0%, 12.5%, and 0% in groups A, B, and C, respectively (Fig. 5; *P* < 0.001).

**Table 5:** Peripheral blood markers to predict irAEs induced by nivolumab treatment

	AUC	95% CI	*P* value
CA19-9	0.692	0.560–0.823	0.022
P-CRP	0.680	0.547–0.822	0.032
CRP	0.656	0.512–0.800	0.063
CAR	0.625	0.472–0.779	0.138
Platelet	0.583	0.420–0.745	0.322
CEA	0.569	0.403–0.735	0.423
PLR	0.553	0.391–0.715	0.527
NLR	0.552	0.399–0.704	0.537
LDH	0.547	0.396–0.697	0.576
PNI	0.470	0.315–0.625	0.724
Albumin	0.442	0.270–0.613	0.488

*irAEs* Immune-related adverse events *AUC*, Area under the curve *CI*, Confidence internal *CA 19–9*, Carbohydrate antigen 19–9 *P-CRP*, Platelet count × serum C-reactive protein level multiplier value *CRP*, C-reactive protein *CAR*, C-reactive protein-to-albumin ratio *CEA*, Carcinoembryonic antigen *PLR*, Platelet-to-lymphocyte ratio *NLR*, Neutrophil-to-lymphocyte ratio *LDH*, Lactate dehydrogenase *PNI*, Prognostic nutritional index

### Prognosis according to the combination of CA19-9 and P-CRP

**Fig. 6:**
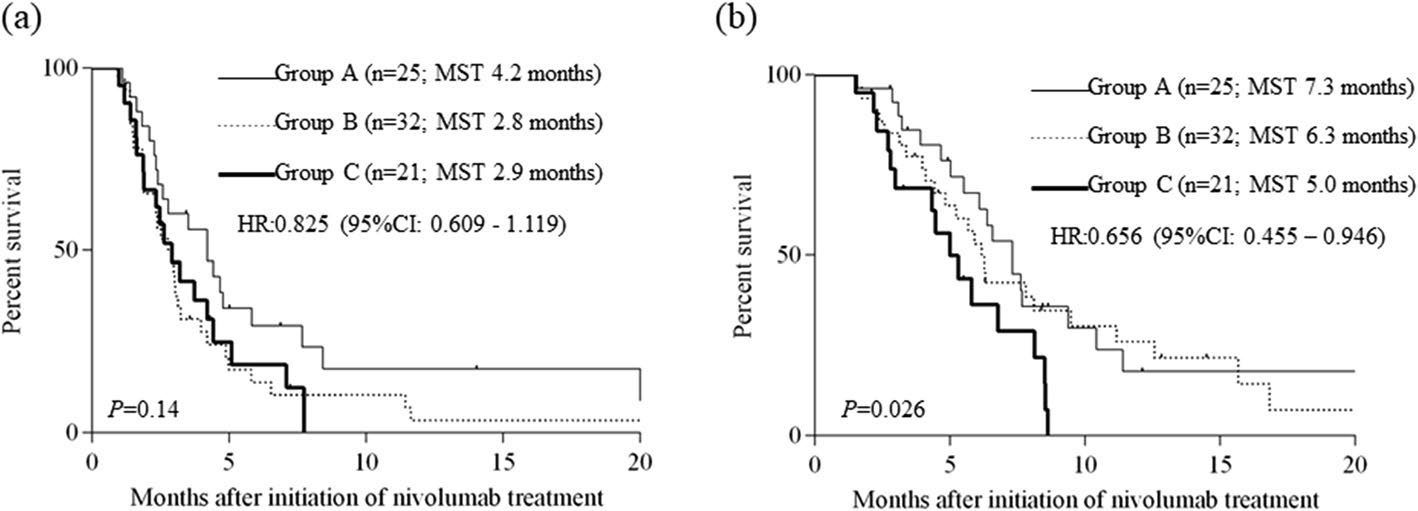
Progression-free survival curves (**a**) and overall survival curves (**b**) according to the combination of CA19-9 and P-CRP Group A, both CA19-9^Low^ and P-CRP^Low^; Group B, either CA19-9^High^ or P-CRP^High^; Group C, both CA19-9^High^ and P-CRP^High^; CA19-9, carbohydrate antigen 19–9; irAEs, immune-related adverse events; MST, median survival time; P-CRP, platelet count × serum C-reactive protein level multiplier value; ROC, receiver operating characteristic

Finally, we determined the prognosis of RUGC patients according to the combination of CA19-9 and P-CRP. The median PFS were 4.2 months, 2.8 months, and 2.9 months in groups A, B, and C, respectively (Fig. 6a). Although the median PFS of group A was longer than that of group B and C, the difference was no statistically significant (*P* = 0.14). The median OS were 7.3 months, 6.3 months, and 5.0 months in groups A, B, and C, respectively (Fig. 6; *P* = 0.026), indicating that the combination of CA19-9 and P-CRP was also useful in predicting the overall survival in RUGC patients who underwent nivolumab treatment.

## Discussion

In this study, we first demonstrated the real world efficacy of nivolumab treatment in RUGC patients. The study results, including ORR, DCR, PFS, and OS, in RUGC patients treated with nivolumab were similar to those reported in the ATTRACTION 2 study [6], confirming that nivolumab treatment for RUGC patients was also effective in a routine clinical setting. Another important study finding was the frequency of patients in whom nivolumab treatment was not effective. PD was observed in approximately 50% of RUGC patients treated with nivolumab, which is the same as found in a previous report [6]. Recent studies have reported an increased frequency of HPD after nivolumab treatment in various types of cancer [19–21]. In GC, Sasaki et al. reported that 21% of advanced GC patients developed HPD after nivolumab treatment [9]. HPD is also observed in patients treated with conventional chemotherapy. In this regard, Aoki et al. reported that HPD was observed more frequently after nivolumab than after irinotecan, an observation that was associated with a poor prognosis after nivolumab but not so clearly after irinotecan [22]. These findings indicated that nivolumab treatment is potentially harmful for such patients. There are other treatment options, including trifluridine/tipiracil [10]. Considering that nivolumab treatment is so expensive, it is extremely important to predict the efficacy of nivolumab treatment before its initiation in RUGC patients. Several predictors of the efficacies of ICPIs treatment, including nivolumab, have been reported thus far, including PD-L1 expression, microsatellite instability, and tumor mutation burden [23–25]. Since those predictors are complicated, time-consuming, and expensive, they are difficult to use in routine clinical settings.

We also demonstrated that irAEs were observed in 15 (19.2%) patients in this study, which was similar to the findings of previous reports [13, 26]. The irAEs are believed to be induced by increased T-cell activation and proliferation caused by nivolumab treatment, which can also cause hyper-autoimmune reactions in some organ systems [27]. Accumulating evidence has shown that irAEs were closely associated with favorable prognosis in various types of cancer. In GC patients, Masuda et al. retrospectively reviewed the outcomes in 65 patients with advanced GC and showed that the survival rate was significantly better in patients with irAEs than in those without. Furthermore, Namikawa et al. reported that patients with irAEs correlated with better OS in patients with GC [14]. We also reported a close correlation between irAEs and favorable prognosis in this study, indicating that irAEs were a useful prognostic indicator in RUGC patients treated with nivolumab. However, it is impossible to predict which patients will have irAEs before initiation of nivolumab treatment. Furthermore, prediction of irAEs before initiation of nivolumab treatment was important for both prediction of prognosis and management of irAEs, which are sometimes life-threatening and require management by a specialist. Therefore, we determined the predictors of irAEs in RUGC patients treated with nivolumab in this study. Since such predictors would be better if they were convenient and minimally invasive for use in routine clinical settings, we focused on indicators that could be obtained from routine blood tests. We found that serum CA19-9 level and P-CRP were good predictive indicators of irAEs. Although previous reports demonstrated that serum LDH level was a useful predictor of prognosis and irAE in some types of cancer [28–31], it was not the case in this study. Pavan et al. reported that PLR, which was believed to be associated with both inflammatory and immune status, was an independent predictive marker of irAEs in advanced non-small-cell lung cancer treated with immune-checkpoint inhibitors [32]. Although PLR was not useful for predicting irAEs in this study, we demonstrated that the P-CRP, which is another platelet-related indicator of inflammation, was a useful predictive indicator of irAEs in this study. The P-CRP has been reported to be a useful prognostic indicator in several cancers, including GC [17, 33, 34]. Inflammatory cytokines, which include interleukin-6 (IL-6), are mediators of tumor-related inflammation [35, 36]. IL-6 can lead to elevation of CRP, which is an acute-phase reactant synthesized by hepatocytes and one of the most frequently used serum markers in evaluating inflammatory status [37]. IL-6 also elevates peripheral PC because IL-6 elicits differentiation of megakaryocytes to platelets [38]. Since the P-CRP was defined as the product of the peripheral thrombocyte count × the serum CRP level, it reflects inflammatory status, which might be closely associated with irAEs.

We also demonstrated that CA19-9 was a useful predictor of irAEs. CA19-9 is a modified Lewis(a) blood group antigen. Since some cancer cells secrete CA19-9, serum CA19-9 level is often elevated in cancer patients. The CA19-9 level is currently recommended for clinical use by the National Comprehensive Cancer Network guidelines for pancreatic cancer and also used as a tumor marker in GC patients. Considering the origin of CA19-9, elevated serum CA19-9 level is likely to reflect tumor burden in cancer patients. We have previously demonstrated that GC cells induced impairment of T-cell function [39]. Therefore, cell-mediated immunity is likely to be more suppressed in patients with elevated serum CA19-9 level than in other patients and might be associated with a low frequency of irAEs. Since the correlation between P-CRP and CA19-9 was relatively weak, we speculated that predicting irAEs might be more useful for the combination of P-CRP and CA19-9 than for either P-CRP or CA19-9 alone. In fact, the AUC of the combination of P-CRP and CA19-9 was much higher than that of either P-CRP or CA19-9 alone, confirming that our speculation was correct.

Nivolumab has a wide therapeutic index, with doses from 0.1 to 10 mg/kg every 2 weeks (Q2W) found to be well tolerated based on early phase dose-ranging data [8, 40]. Nivolumab was administered intravenously at a dose of either 3 mg/kg or 240 mg/body every 2 weeks in this study. However, plasma concentration of nivolumab might be related to the onset of irAEs. In this regard, the previous study demonstrated that no pharmacokinetic/pharmacodynamic (PK/PD) relationship was observed with either survival or onset of irAE in non small-cell lung cancer (NSCLC) patients treated with nivolumab [41]. However, there is no data showing the correlation between PLK/PD and onset of irAE in GC patients thus far. Further investigations are warranted to determine the correlation between PLK/PD and onset of irAE in GC patients.

This retrospective study had some limitations. First, this was a retrospective analysis of data from nine hospitals, which probably caused some selection bias. Second, the number of patients included in this study was small; therefore a large-scale study is needed to confirm our results. Third, some proportion of patients could be Lewis's antigen negative. It has been reported that the frequency of Lewis's antigen negative is approximately 5–10% [42]. Since CA19-9 is a modified Lewis(a) blood group antigen, the secretion of CA19-9 is scarce in such patients. Since Lewis's antigen was not determined in this study, further studies are urgently required to confirm our results.

## Conclusions

We demonstrated that the prognosis of RUGC patients was significantly better for those with irAEs than for those without irAEs, in this multicenter study. The combination of CA19-9 and P-CRP might help physicians properly manage irAEs and select appropriate treatment in RUGC patients.

## Data Availability

The datasets used and/or analyzed during the current study are available from the corresponding author on reasonable request.

## Abbreviations

ALB: Albumin
AUC: Area under the curve
CA19-9: Carbohydrate antigen 19–9
CI: Confidence interval
DCR: Disease control rate
ECOG: Eastern Cooperative Oncology Group
GC: Gastric cancer
HPD: Hyperprogressive disease
ICPIs: Immune-check point inhibitors
IL-6: Interleukin-6; irAEs: immune-related adverse events
LC: Lymphocyte count
LDH: Lactate dehydrogenase
MST: Median survival time
NC: Neutrophil count
ORR: Objective response rate
OS: Overall survival
PC: Platelet count
P-CRP: PC × serum CRP level multiplier value
PD: Programmed cell death
PD: Progressive disease
PFS: Progression-free survival
PLR: Platelet-to-lymphocyte ratio
PNI: Prognostic nutritional index
PS: Performance status
RECIST: Response Evaluation Criteria in Solid Tumors
ROC: Receiver operating characteristic
RUGC: Recurrent or unresectable advanced gastric cancer
